# Two novel *PCDH19* mutations in Russian patients with epilepsy with intellectual disability limited to females: a case report

**DOI:** 10.1186/s12881-020-01119-6

**Published:** 2020-10-21

**Authors:** Anastasiya Aleksandrovna Kozina, Elena Grigorievna Okuneva, Natalia Vladimirovna Baryshnikova, Inessa Dmitrievna Fedonyuk, Alexey Aleksandrovich Kholin, Elena Stepanovna Il’ina, Anna Yurievna Krasnenko, Ivan Fedorovich Stetsenko, Nikolay Alekseevich Plotnikov, Olesia Igorevna Klimchuk, Ekaterina Ivanovna Surkova, Valery Vladimirovich Ilinsky

**Affiliations:** 1 Institute of Biomedical Chemistry, Pogodinskaya street 10/8, 119121 Moscow, Russia; 2 Pirogov Russian National Research Medical University, Ostrovitianova street 1, 117997 Moscow, Russia; 3 Genotek Ltd., Nastavnicheskii pereulok 17/1, 105120 Moscow, Russia; 4 Russian Children’s Clinical Hospital of Pirogov Russian National Research Medical University, Leniskiy prospekt 117, 117513 Moscow, Russia; 5 Vavilov Institute of General Genetics, Gubkina street 3, 119333 Moscow, Russia

**Keywords:** Epilepsy with intellectual disability limited to females, EIEE9, *PCDH19*, Protocadherin 19, Case report

## Abstract

**Background:**

Epilepsy with intellectual disability limited to females (Epileptic encephalopathy, early infantile, 9; EIEE9) is a rare early infantile epileptic encephalopathy characterized by an unusual X-linked inheritance: females with heterozygous mutations are affected, while hemizygous males are not.

**Case presentation:**

We describe the clinical and molecular characteristics of 2 Russian patients with EIEE9 (females, ages 3 years and 7 years). In these patients seizures developed at the age of 3 years. Additionally, for our patients and for cases described in the literature we searched for a possible relationship between the type and localization of the mutation and the EIEE9 clinical phenotype.

**Conclusions:**

We identified two novel *PCDH19* mutations in EIEE9 patients: a missense mutation in exon 1 (c.1236C > A, p.Asp412Glu) and a frameshift in exon 3 (c.2386_2387insGTCT, p.Thr796fs). We conclude that the age of seizure onset and the presence of intellectual disability may depend not on the type and localization of *PCDH19* mutations, but on the X-inactivation status. The study also highlights the need to screen for EIEE9 among young female epilepsy patients.

## Background

Epilepsy with intellectual disability limited to females (early infantile epileptic encephalopathy type 9, OMIM 300088, EIEE9) is a rare X-linked disease affecting heterozygous females and sparing hemizygous males. EIEE9 was first described by [1].

The main characteristic of EIEE9 is early infantile (6–36 months) onset of seizures of various types (tonic, clonic, tonic-clonic, partial seizures) and severity [2]. Seizures usually are provoked by fever and are resistant to anti-epileptic drugs [3]. Additionally, patients often have cognitive impairment up to severe intellectual disability [4].

EIEE9 is caused by mutations in the *PCDH19* (protocadherin 19) gene [5]. This gene is located on the X-chromosome, thus EIEE9 has an X-linked pattern of inheritance. Disorders with the same type of inheritance are typically characterized by affected males and unaffected carrier females. However, core clinical symptoms of EIEE9 manifest only in females, while carrier males may have several neuropsychiatric features such as obsessional tendencies [3]. Males with mosaic mutations can also be affected with a phenotype similar to affected females [2, 6, 7]. Some authors explain this unique sex-limited expression pattern of EIEE9 by the mechanism called “cellular interference” either due to X-chromosome inactivation in females or somatic mosaicism in males [8, 9]. However, experimental evidence for the accuracy of this model remains insufficient.

The *PCDH19* gene includes six exons and encodes the 1148-amino-acid (AA) protein. PCDH19 protein belongs to the protocadherin δ2 subgroup of the cadherin superfamily [10]. Protocadherins, including PCDH19, play a role in neuronal migration and formation of synaptic connections in the brain [11, 12]. However, clear cellular functions, molecular mechanisms and signaling partners have yet to be determined. Cooper and colleagues showed that protocadherin-19 molecules from adjacent cells form adhesive connections between neurons. Some of the mutations that cause epilepsy occur in the region responsible for what Cooper et al. characterize as a “forearm handshake” [13].

Dibbens et al. were first to identify mutations in the *PCDH19* gene in seven families with EIEE9 [5]. To date, more than 150 mutations in *PCDH19* have been revealed. *PCDH19* is currently considered the second most clinically significant gene for epilepsy after *SCN1A*. EIEE9-associated *PCDH19* mutations can be missense or have a truncating functional effect (nonsense, frameshift, splicing) [2]. Most of these mutations are de novo mutations in exon 1, which encodes the entire extracellular domain of protocadherin 19. No mutations were found in exon 2 and mutations are extremely rare in exons 3–6 [2, 8, 14, 15].

Here, we describe clinical and genetic characteristics of two Russian EIEE9patients. We identified two novel *PCDH19* mutations in these patients.

## Case presentation

The study conforms to the Declaration of Helsinki. All research was approved by the ethics committee of Genotek Ltd. (05/2019). The patients’ parents provided written informed consent to studies and publication of clinical information and sequencing data.

Among all of the families there was no history of seizures or intellectual disability. All patients were diagnosed with EIEE9 when *PCDH19* (NM_001184880.2) mutations were found.

### Patient 1 (3 years old, female, 23 kg, 103 cm)

The pregnancy and perinatal history were unremarkable. The girl developed without abnormalities to the age of 3 years. At 3 years the girl developed generalized onset motor tonic-clonic seizures with apnoea at night against a background of fever. A month later the seizures were repeated against the background of normal temperature. Seizures during wakefulness first developed at 3 years and 2 months. Seizures persisted despite treatment with valproic acid.

Intelligence was normal. Speech was restricted to phrases. Ophtalmologic examination revealed no pathology. Ultrasound examination revealed hepatosplenomegaly. A blood test showed elevated lactate level (2,6 mmol/L, normal < 1,7 mmol/L).

**Fig. 1:**
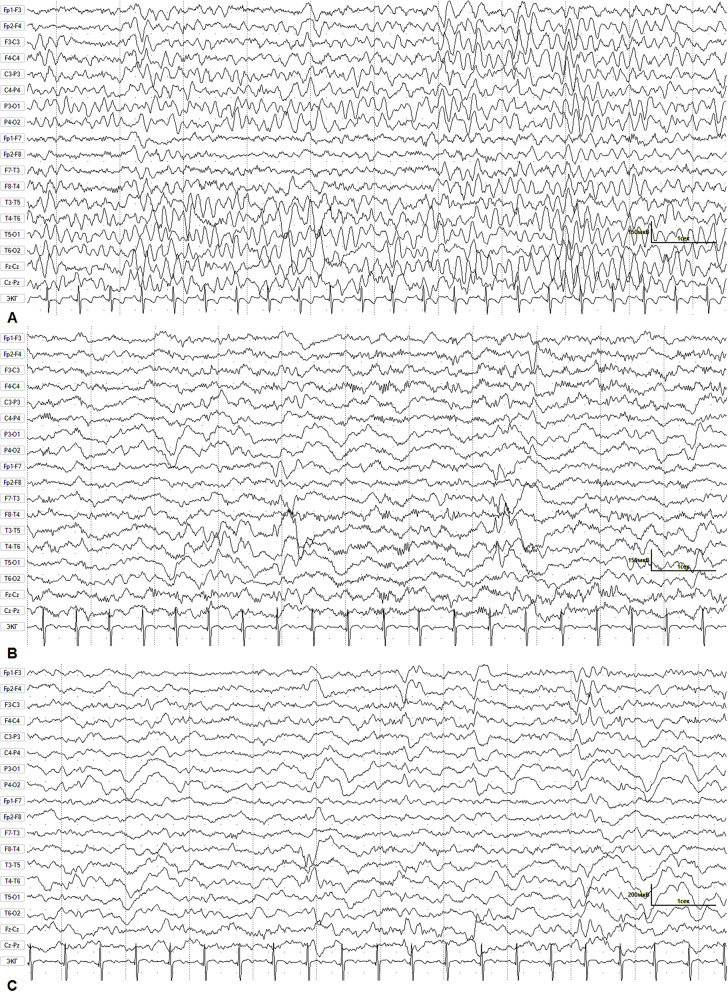
EEG for patient 1 during wakefulness and sleep. **a**. Waking EEG. Rhythmic theta-activity with tendency to hypersynchronization. Regional delta-slow accentuation in right temporal region with polyphasic potentials, but with no typical epileptiform activity at wake state. **b**. Sleep EEG. Regional epileptiform activity – sharp-and-slow wave discharges in the left temporal region. Moderate fusiform brush-like beta-activity accentuations in fronto-centro-temporal D > S regions. **c**. Sleep EEG. Regional epileptiform activity – sharp-and-slow wave discharges in the right temporal region. Preserved sleep architectonics with the presence of physiological sleep patterns (K-complexes and “sleep spindles”)

Video electroencephalogram (VEEG) was performed for 2.5 h in states of active and passive wakefulness, sleep, and during functional tests (Fig. 1). The basic rhythm was represented by a regular, stable, modulated alpha rhythm with a frequency of 7–8 Hz and an amplitude up to 70 μV. This rhythm was recorded in the occipital regions with distribution to the posterior temporal and parietal regions of the hemispheres. Rhythmic theta-activity with tendency to hypersynchronization and regional delta-slow accentuation in the right temporal region with polyphasic potentials, but with no typical epileptiform activity were revealed at wake state. During rhythmic photostimulation, a rhythm following reaction was not observed. Photoparoxysmal response has not been recorded. A 3-min hyperventilation test revealed a moderate disorganization of cortical rhythm in the form of diffuse slow-wave activity of the theta range without a significant increase in amplitude. At the end of the test, there was rapid restoration of background. Sleep was modulated in phases; physiological patterns of sleep were differentiated. During sleep, regional epileptiform activity in the form of sharp-and-slow wave discharges in the temporal regions was revealed. K-complexes in the form of high-amplitude flashes of slow biphasic and polyphase waves with an amplitude of up to 190 μV were periodically recorded.

The brain magnetic resonance imaging (MRI) revealed a region of gliosis in the left frontal lobe.

The patient received antiepileptic treatments (valproic acid 600 mg/day, topiramate 25 mg/day, levetiracetam 1000 mg/day) without significant improvement.

### Patient 2 (7 years old, female, 26 kg, 130 cm)

The pregnancy was complicated by a threat of preterm birth at 16 weeks and chlamydia. Up to 3 years and 2 months, the girl developed according to her age with periodic tension of the neck muscles which started at 7 months. At the age of 3 years and 2 months the first febrile seizure occurred in the form of tonic tension of the extremities at night (generalized onset motor tonic-clonic seizures). Seizures persisted despite treatment with valproic acid (500 mg/day). Clusters of seizures repeated every 5–10 days. Aggression and hysterical reactions were observed after the seizures. However, intellectual abilities were age appropriate. Ophthalmologic examination revealed no pathology.

**Fig. 2:**
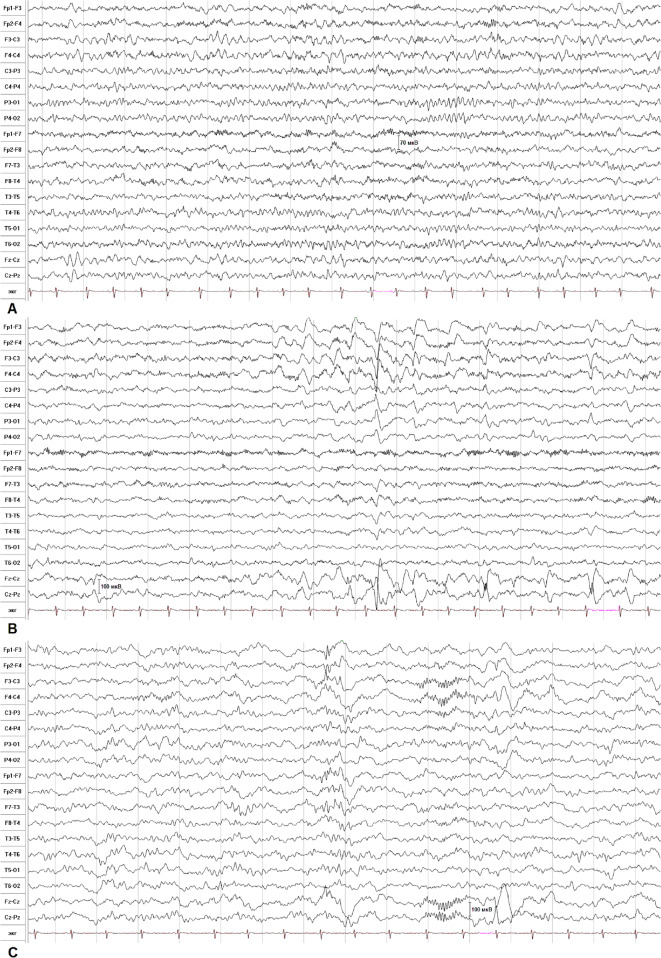
EEG of patient 2 during wakefulness and sleep. **a**. Waking EEG. Physiological 8–9,5 Hz alpha rhythm in the posterior regions. Rhythmic 5–6 Hz theta activity in frontal regions and theta accentuation in the vertex region. **b**. Sleep EEG. Moderate fusiform brush-like beta-activity accentuations in frontal (predominantly left frontopolar-inferior frontal regions). No epileptiform activity was identified. **c**. Sleep EEG. Atypical K-complexes with spike-like insertions in frontal regions. No typical epileptiform spike-wave discharges were identified

The VEEG was performed for 2.5 h in states of active and passive wakefulness, sleep, and functional tests (Fig. 2). The basic rhythm was represented by a regular, stable, modulated alpha rhythm with a frequency of 8–9,5 Hz. Rhythmic 5–6 Hz theta activity in the frontal regions and theta accentuation in the vertex region, but with no typical epileptiform activity were revealed at wake state. Sleep was modulated in phases; physiological patterns of sleep were differentiated (K-complexes, vertex-potentials, sleep spindles). Moderate fusiform brush-like beta-activity accentuations and atypical K-complexes with spike-like insertions were revealed in frontal regions.

With antiepileptic (valproic acid 600 mg/day, levetiracetam 500 mg/day) and antipsychotic (aminophenylbutyric acid 250 mg/day) treatment at the time of analysis, seizures were not observed.

### Genetic studies

After obtaining informed consent for the genetic analyses, targeted exome enrichment and sequencing were performed using patient and parental genomic DNA extracted from circulating leukocytes.

### Exome sequencing

Exome sequencing of samples was carried out by Genotek Ltd. Genomic DNA from peripherial blood samples was extracted using QIAamp DNA Mini Kit (Qiagen, Hilden, Germany) according to the manufacturerʼs protocol. DNA libraries were prepared using the QIAseq FX DNA library kit (Qiagen, Hilden, Germany). Target enrichment, sequencing and quality control were performed as described previously [16]. To estimate pathogenicity, data were extracted from the dbNSFP, Clinvar, OMIM, and HGMD databases. The variants were analyzed in silico using Scale-Invariant Feature Transform (SIFT) and Polymorphism Phenotyping v2 (PolyPhen-2). Mutant allele frequencies were extracted from the 1000Genomes, ExAC, and Genotek databases. Pathogenicity was evaluated in accordance with international recommendations of ACMG (American College of Medical Genetics and Genomics), CAP (College of American Pathologists), and AMP (Association for Molecular Pathology).

### Sanger sequencing

All variants found by exome sequencing were confirmed by Sanger sequencing. Additionally, we used Sanger sequencing to confirm the presence of *PCDH19* mutations in the parents of probands. BigDye Terminator Cycle Sequencing Kit v3.1 (Thermo Fisher Scientific) was used to label amplicons with fluorescent labels. Sanger sequencing was performed on ABI PRISM 3500 Genetic Analyzer (Applied Biosystems) in accordance with the protocol of manufacturer.

All described variants are under consideration in the ClinVar database.

## Discussion and conclusions

We identified two novel heterozygous mutations in the *PCDH19* gene (NM_001184880.2) in two girls.

**Fig. 3:**
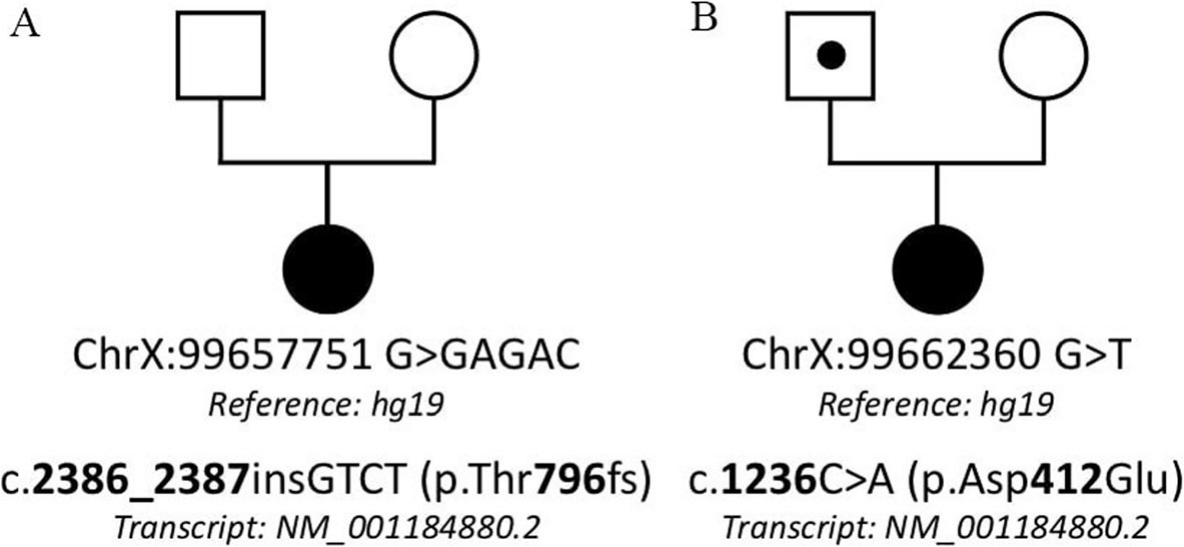
Pedigrees of Russian families with EIEE9. **a**: proband - patient 1 with heterozygous de novo *PCDH19* mutation c.2386_2387insGTCT (p.Thr796fs). **b**: proband – patient 2 with heterozygous *PCDH19* mutation c.1236C > A (p.Asp412Glu). The proband has inherited this mutation from the father. Black circle: affected mutation-carrying female; white circle: female without mutation; white square: male without mutation; dot within a white square: asymptomatic mutation-carrying male

The c.2386_2387insGTCT mutation was not previously reported in literature and databases. This mutation is an insertion of 4 nucleotides in exon 3 and is predicted to have caused a frameshift mutation at position 796 of the amino acid sequence. The mutation led to a change from Thr to Ser at position 796 and a translation termination codon at position 19 of the new reading frame(p.Thr796SerfsTer19). Using the secondary structure of *PCDH19*, we revealed that this mutation is located in the cytoplasmic domain and produces a truncated protein lacking the intracellular domain. According to the ACMG criteria, this mutation can be classified as pathogenic (PVS1, PS2, PM2, PP3). The c.2386_2387insGTCT mutation is absent in parents of the proband (de novo) (Fig. 3).

The c.1236C > A (p.Asp412Glu) mutation was not previously reported in literature and databases. This missense mutation results in a single amino acid change in a highly conserved extracellular protein domain. At the molecular level, this results in a negatively charged Glutamic acid instead of a negatively charged Aspartic acid. This change does not potentially impair the electrostatic interaction between the amino acid residues and does not destabilize the β-chain structure. AA substitutions in the cytoplasmic domain appear to have less or no deleterious effects on protein function. According to the ACMG criteria, this mutation can be classified as likely pathogenic (PM1, PM2, PP3, PP4). The mutation was inherited from the paternal line. The girl’s father was identified to be a hemizygote while the mother did not carry this variant (Fig. 3). But the father of patient 2 is healthy and had no seizures. There is no family history of epilepsy on the father’s side.

The *PCDH19* mutations cause EIEE9, however, the disease-causing mechanisms are not fully understood. It has been hypothesized that the mosaicism of normal *PCDH19* and mutant-expressing cells results in “scrambling” of the neuronal brain circuitry in affected people (cellular interference).

The phenotype of EIEE9 can be different – girls with the disorder have symptoms ranging from benign focal epilepsy with normal cognitive abilities to severe seizures and intellectual disability similar to Dravet syndrome.

PCDH19 is highly conserved across human, mouse, zebrafish, and chicken and has weak homophilic cell adhesion characteristics. It has been shown that PCDH19 interacts with N-cadherin and members of the WAVE complex (Nap1 and Cyfip2) suggesting that each has a role in controlling cell movements during embryogenesis [17, 18]. The majority of the *PCDH19* mutations were observed in a highly conserved extracellular protein domain (AA 1–678) that is essential for the normal function of cadherin [13]. These mutations are mostly missense. Truncating mutations have been found in all domains of the PCDH19 protein [15].

We identified two novel *PCDH19* mutations in EIEE9 patients. We compared the clinical and genetic characteristics of these patients with those described previously (with different age of seizures and level of intellectual disability) (Table 1) [19–23]. We expected to detect early onset of seizures and intellectual disability only in patients with mutations in exon 1, as it is important for the interaction of protocadherin with other molecules. However, an analysis of the data did not confirm this. We found that the severity of EIEE9 do not correlate with the localization and type of the *PCDH19* mutation. The different level of intellectual disability in the described EIEE9 patients can be explained by the random nature of X inactivation in the different parts of the brain.

**Table 1:** Comparison of *PCDH19* mutations identified in this study (bold) and previously described *PCDH19* mutations in females with EIEE9

Mutation	Exon	Type	Onset of seizures, months	Intelligence	Source
c.445C > T p.Pro149Ser	1	Missense	17	normal	[19]
c.695A > G (p.Asn232Ser)	1	Missense	4	intellectual disability	[20]
c.918C > G, p.Tyr306*	1	Nonsense	20	normal	[21]
c.937G > A p.Glu307Lys	1	Missense	70	normal	[19]
c.919G > A p.Glu313Lys	1	Missense	32	normal	[19]
c.1183C > T (p.Arg395*)	1	Nonsense	8	intellectual disability	[22]
**c.1236C > A (p.Asp412Glu)**	1	Missense	38	normal	This study (Patient 2)
c.1681C > T, p.Pro561Ser	1	Missense	14	normal	[21]
c.1765_1766delTG p.Val589CysfsX8	1	Frameshift	9	intellectual disability	[19]
**c.2386_2387insGTCT (p.Thr796fs)**	3	Frameshift	36	normal	This study (Patient 1)
c2468delT, p.L823fs	3	Frameshift	18	intellectual disability	[23]

We also confirmed the suggestion of Kolc and colleagues that age at seizure onset determines the prognosis of the disease. An early onset of seizures (≤12 months) leads to more severe intellectual disability as compared to onset at> 12 months [2].

Genetic testing could provide direct evidence of mutations associated with EIEE9. This analysis is a rapid and practical tool to confirm the diagnosis of EIEE9. Genetic testing should be considered for young female patients with infantile onset seizures and developmental delay.

## Data Availability

The datasets generated and analyzed during the current study are available in the Genotek repository, gf1232.1680CC36C.1.fastq.gzgf1232.1680CC36C.2.fastq.gztw8628.594DA854D.1.fastq.gztw8628.594DA854D.2.fastq.gzGRCh37.p13 genome assembly (https://www.ncbi.nlm.nih.gov/assembly/GCF_000001405.25/).

## Abbreviations

ACMG: American College of Medical Genetics and Genomics
AMP: Association for Molecular Pathology
CAP: College of American Pathologists
EIEE9: Epileptic encephalopathy, early infantile, 9

## References

[1] Juberg RC, Hellman CD (1971). A new familial form of convulsive disorder and mental retardation limited to females. J Pediatr.

[2] Kolc KL, Sadleir LG, Scheffer IE (2019). A systematic review and meta-analysis of 271 PCDH19-variant individuals identifies psychiatric comorbidities, and association of seizure onset and disease severity. Mol Psychiatry.

[3] Scheffer IE, Turner SJ, Dibbens LM (2008). Epilepsy and mental retardation limited to females: an under-recognized disorder. Brain..

[4] Liu A, Xu X, Yang X (2017). The clinical spectrum of female epilepsy patients with PCDH19 mutations in a Chinese population. Clin Genet.

[5] Dibbens LM, Tarpey PS, Hynes K (2008). X-linked protocadherin 19 mutations cause female-limited epilepsy and cognitive impairment. Nat Genet.

[6] de Lange IM, Rump P, Neuteboom RF (2017). Male patients affected by mosaic PCDH19 mutations: five new cases. Neurogenetics..

[7] Liu A, Yang X, Yang X (2019). Mosaicism and incomplete penetrance of *PCDH19* mutations. J Med Genet.

[8] Depienne C, LeGuern E (2012). PCDH19-related infantile epileptic encephalopathy: an unusual X-linked inheritance disorder. Hum Mutat.

[9] Perez D, Hsieh D, Rohena L (2017). Somatic mosaicism of PCDH19 in a male with early infantile epileptic encephalopathy and review of the literature. Am J Med Genet A.

[10] Morishita H, Yagi T (2007). Protocadherin family: diversity, structure, and function. Curr Opin Cell Biol.

[11] Cooper SR, Emond MR, Duy PQ (2015). Protocadherins control the modular assembly of neuronal columns in the zebrafish optic tectum. J Cell Biol.

[12] Emond MR, Biswas S, Jontes JD (2009). Protocadherin-19 is essential for early steps in brain morphogenesis. Dev Biol.

[13] Cooper SR, Jontes JD, Sotomayor M (2016). Structural determinants of adhesion by Protocadherin-19 and implications for its role in epilepsy. Elife..

[14] Depienne C, Trouillard O, Bouteiller D (2011). Mutations and deletions in PCDH19 account for various familial or isolated epilepsies in females. Hum Mutat.

[15] van Harssel JJ, Weckhuysen S, van Kempen MJ (2013). Clinical and genetic aspects of PCDH19-related epilepsy syndromes and the possible role of PCDH19 mutations in males with autism spectrum disorders. Neurogenetics..

[16] Kozina AA, Trofimova TA, Okuneva EG (2019). Liddle syndrome due to a novel mutation in the γ subunit of the epithelial sodium channel (ENaC) in family from Russia: a case report. BMC Nephrol.

[17] Emond MR, Biswas S, Blevins CJ, Jontes JD (2011). A complex of Protocadherin-19 and N-cadherin mediates a novel mechanism of cell adhesion. J Cell Biol.

[18] Tai K, Kubota M, Shiono K, Tokutsu H, Suzuki ST (2010). Adhesion properties and retinofugal expression of chicken protocadherin-19. Brain Res.

[19] Leonardi E, Sartori S, Vecchi M (2014). Identification of four novel PCDH19 mutations and prediction of their functional impact. Ann Hum Genet.

[20] Smith L, Singhal N, El Achkar CM (2018). PCDH19-related epilepsy is associated with a broad neurodevelopmental spectrum. Epilepsia..

[21] Tan Y, Hou M, Ma S (2018). Chinese cases of early infantile epileptic encephalopathy: a novel mutation in the PCDH19 gene was proved in a mosaic male- case report. BMC Med Genet.

[22] Marini C, Darra F, Specchio N (2012). Focal seizures with affective symptoms are a major feature of PCDH19 gene-related epilepsy. Epilepsia..

[23] Zhang X, Chen N, Ma A (2018). Case report of a novel PCDH19 frameshift mutation in a girl with epilepsy and mental retardation limited to females. Medicine (Baltimore).

